# The Effect of Different Feeding Applications on the Swimming Behaviour of Siberian Sturgeon: A Method for Improving Restocking Programmes

**DOI:** 10.3390/biology10111162

**Published:** 2021-11-10

**Authors:** Tatyana Gebauer, Radek Gebauer, Petr Císař, Hung Quang Tran, Ondřej Tomášek, Peter Podhorec, Markéta Prokešová, Alexander Rebl, Vlastimil Stejskal

**Affiliations:** 1 South Bohemian Research Center of Aquaculture and Biodiversity of Hydrocenoses, Faculty of Fisheries and Protection of Waters, Institute of Aquaculture and Protection of Waters, University of South Bohemia in Ceske Budejovice, Husova tř. 458/102, 370 05 České Budějovice, Czech Republic; rgebauer@frov.jcu.cz (R.G.); htranquang@frov.jcu.cz (H.Q.T.); ondra.tomasek@gmail.com (O.T.); podhorec.peter@seznam.cz (P.P.); mprokesova@frov.jcu.cz (M.P.); stejskal@frov.jcu.cz (V.S.); 2 Institute of Genome Biology, Research Institute for Farm Animal Biology (FBN), Wilhelm-Stahl-Allee 2, 18196 Dummerstorf, Germany; 3 Laboratory of Signal and Image Processing, Institute of Complex Systems, Faculty of Fisheries and Protection of Waters, CENAKVA, University of South Bohemia in Ceske Budejovice, Zámek 136, 373 33 Nové Hrady, Czech Republic; cisar@frov.jcu.cz

**Keywords:** *Acipenser baerii*, day/night feeding regimes, modified swimming behaviour, predator-free environment, starvation, recirculating aquaculture system

## Abstract

**Simple Summary:**

Restocking programmes are of crucial importance for the stabilisation of wild sturgeon stocks. Despite massive restocking, the survival rate of post-released fish is very low. The low survival is most likely caused by poor fish adaptability due to hatchery rearing practices. This study aimed to provide a deeper understanding of the effect of different feeding applications with day and night regimes and periods of starvation on the modified swimming behaviour of Siberian sturgeons reared in recirculating aquaculture conditions. Our data showed that the modified behaviour of the Siberian sturgeon in our study was caused by fish starvation rather than by the method of feed application or day/night light regimes.

**Abstract:**

Restocking programmes of different fish species have been implemented worldwide. However, the survival of hatchery-reared fish after release to riverine ecosystems is at a very low level. One of the reasons for the high mortality rate of post-released fish is their modified swimming behaviour due to the hatchery rearing practice. To investigate one of the possible causes for modified swimming behaviour, *Acipenser baerii* larvae were exposed to surface- and bottom-feeding applications with day and night light regimes in a factorial design. We also analysed the effect of 5 and 10 days of starvation after different feeding applications on sturgeon swimming behaviour. The surface-feeding application was previously expected to promote the frequent Siberian sturgeon swim up to the mid- and top-water layers in our rearing facilities. However, our results indicated that the modified behaviour of the Siberian sturgeon in our study was caused by fish starvation and a possible predator-free environment rather than by the method of feed application or the day/night light regimes. These results may be used to improve the implementation of restocking programmes either through modified hatchery rearing practice or the training of foraging skills with predator stimuli.

## 1. Introduction 

The exponential growth in the agriculture and food industry [1,2,3] has been attributed to the ever-increasing consumption of freshwater fish [4]. Over the last few decades, freshwater environments, and particularly riverine ecosystems, have undergone vast modifications, including river fragmentation (damming), pollution, and habitat alteration [5,6,7]. These modifications have changed the structure of spawning sites or impeded the migration of rheophilic fish species to spawning areas, which eventually contributed to severely declined populations [8,9,10,11]. This is of particular concern for anadromous/catadromous fish species, including sturgeons [12,13,14]. Further, sturgeons are subjected to massive exploitation by legal and illegal fisheries, since the demand for highly valued sturgeon meat and caviar is continuing to be on a high level [15,16]. Unsustainable stocking measures have also increased the pressure on the remaining natural populations [17].

Various regional, national, and international organisations aim to slow down the dramatic decline in sturgeon populations [16,18,19]. The Danube Delta Biosphere Reserve has been regulating sturgeon fisheries since 1991 [20]. The development of sturgeon aquaculture has reduced the fishing pressure on natural stocks and satisfied the market, lowering prices and making illegal trades less attractive [17,21,22,23]. Restocking programmes are of crucial importance for the stabilisation of wild sturgeon stocks [24,25,26]. Despite massive restocking actions, the sturgeon populations have not recovered significantly [27,28,29,30,31]. This is most likely caused by poor adaptability, which impairs the survival of hatchery-reared fish in the wild [32,33]. For example, more than 250 million starry sturgeons and 17 million Russian sturgeon larvae were released in the Caspian Sea during the period 1902–1941, but the recorded survival rate of released stocks was very low [34]. The same applies to the released stocks of beluga *Huso huso*, the Persian sturgeon *Acipenser persicus* [35], and other fish species [36,37].

The low survival rate of hatchery-reared fish in the wild may origin in specific feeding and swimming behaviours linked to feeding protocols in mass-rearing facilities. For example, a surface-feeding regime has been suggested to cause the high mortality of hatchery-reared benthic fish in the first few days after release to the wild [38,39]. The application of the feed, mainly commercial pellets that float motionlessly on the surface, habituates the fish to the behavioural pattern of the ‘off-bottom swimming activity’. In the wild, this behavioural pattern increases the risk of falling prey to predators [40,41,42]. Different feed applications have an obvious effect on the swimming behaviour of benthic feeders. Many cases of surface and inverted swimming patterns have also been recorded in juveniles of green sturgeon *Acipenser medirostris* [43], shovelnose sturgeon *Scaphirhynchus platorynchus*, Atlantic sturgeon *Acipenser oxyrhynchus* [44], and Siberian sturgeon *Acipenser baerii* kept in captivity. The sturgeons’ modified swimming patterns could also be affected by periods of starvation. This was shown for the reared larvae of Chinese sturgeon *Acipencer sinensis* and shortnose sturgeon *Acipenser brevirostrum*, where the fish increased their swimming activity during starvation for food searching [45,46]. The increased swimming activity made larvae less responsive to predators and easily susceptible to falling to prey [46]. To date, studies on the potential relationship between the application of feed regimes and swimming behaviour of Siberian sturgeons in hatcheries are deficient.

This study exposed Siberian sturgeons to two feeding applications (surface and bottom) with day and night feeding regimes in a factorial design. We also analysed the effect of starvation after different feeding applications on swimming behaviour. 

## 2. Materials and Methods

### 2.1. Fish Acquisition and Husbandry

Larvae of Siberian sturgeons (4 dph; initial body weight 0.04 ± 0.01 g; total length 17.1 ± 0.7 mm) were obtained from the hatchery Fischzucht Rhönforelle GmbH & Co. KG, Gersfeld, Germany. Fish were transported by car in oxygenated polyethylene bags (filled with 1/3 water, 2/3 oxygen) placed in a thermobox to the experimental facility of the Faculty of Fisheries and Protection of Waters, the University of South Bohemia, in České Budějovice, Czech Republic.

After gradual (1 °C per hour) water temperature acclimatisation from 11 to 19 °C, the larvae were housed in a density of 32 individuals L^−1^ in six white rectangular tanks (85 l × 23 w × 34 h cm; net water volume 50 L) with a water exchange rate of 84 L (equates 3.5 tanks per hour). The concentration of oxygen (above 90% of saturation), pH (7.0 ± 0.3), and temperature (19.0 ± 1 °C) were monitored twice daily throughout the experiment with a multimetre (Hach Lange HQ40d, Düsseldorf, Germany). Concentrations of ammonium (0.05 ± 0.01 mg L^−1^) and nitrites (0.05 ± 0.02 mg L^−1^) were measured every second day with a portable spectrophotometer (DR 2800, Hach Company, Loveland, CO, USA). The light intensity was set at 250 lx at the water surface (DT-8809, Cem, Hong Kong, China), and the photoperiod was constant at 12L:12D.

With the onset of exogenous feeding (10–12 dph), the larvae were fed five times daily ad libitum with a mixture of *Artemia nauplii* (size of 430–460 μm, hatching rate above 260,000 nauplii g^−1^; Ocean Nutrition, France) and chopped live tubificid worms (*Tubifex tubifex*) at a 50:50 ratio for the first 10 days of exogenous feeding. Afterward, the larvae were progressively weaned on commercial pelleted food (Nutra 0.5-mm, Skretting, Norway) over a period of three days (20–23 dph) and were manually fed at two-hour intervals from 8 a.m. to 6 p.m. 

### 2.2. Feeding Regimes Adaptation

**Figure 1:**
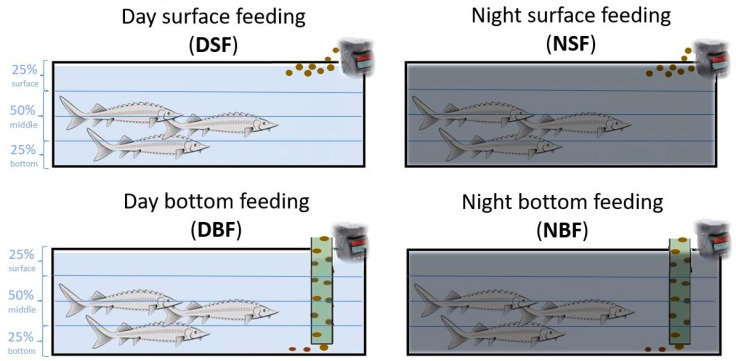
Experimental setup of the two feeding applications (surface and bottom) during day- and night-time hours. Lines show the three video-recorded tank sections: 25% bottom, 50% middle, and 25% surface.

On 23 dph, the larvae were randomly redistributed in 12 tanks (85 l × 23 w × 34 h cm; net water volume 50 L, with a water exchange rate of 1.6 tanks per hour) in a density of 5.7 individuals L^−1^. Four feeding regimes were applied in triplicate: (i) DSF, surface feeding at day; (ii) NSF, surface feeding at night; (iii) DBF, bottom-feeding at day; (iv) NBF, bottom-feeding at night (Figure 1). The larvae were fed with commercial pellets (Nutra, Skretting, Norway) distributed either on the water surface (DSF and NSF) or at the bottom (DBF and NBF) using a funnel 1.5 cm diameter tube. The pellets were provided to the fish by automatic feeders (Eheim, model 3582, Deizisau, Germany) six times and one time by hand daily ad libitum from 8 a.m. to 8 p.m., for day feeding application, and from 8 p.m. to 8 a.m. for night feeding application. At 46 dph, the stocking density of sturgeons was unified to 2.5 individuals L^−1^. Between 68 dph and 120 dph, the swimming behaviour of the sturgeons was video recorded.

### 2.3. Video Recording of Swimming Behaviour

Six acrylic transparent experimental tanks (117l × 20 w × 97 h cm; with net water volume 200 L were monitored using video recording. Tank walls were covered with a white vinyl sheet on the back and two lateral sides to avoid the reflection of fish during recording. Before each video recording, the tanks were filled with dechlorinated tap water (O_2_ ≥ 90%, pH 7.0 ± 0.3, t 19.0 ± 1 °C). Every 3 weeks (i.e., at 68, 89, and 110 dph), 60 fish (20 ind. in triplicate) per feeding group (i.e., DSF, NSF, DBF, NBF) were recorded using a video camera (DS-2CD2043G0-I, Hikvision, Hangzhou, China) placed at a 100 cm distance in front of the tanks. After one hour of adaptation, the fish in the experimental tanks were video recorded for 60 min every four hours (the video recordings from 8:00 a.m., 12:00 noon, 4 p.m., 8 p.m. were referred to as daylight hours, and from 8 p.m. 12:00 midnight, 4:00 a.m., and 8 a.m. were referred to as night hours). Tested fish were not returned to the same tanks of different feeding regimes, nor were they repeatedly used.

### 2.4. Effect of Starvation on Swimming Behaviour after Different Feeding Applications

To analyse the behaviour of sturgeon after a starvation period, the tested individuals (i.e., 60 randomly chosen fish (20 ind. per triplicate) for feeding group DSF, NSF, DBF, and NBF were taken from the husbandry tanks described in Section 2.2) were placed into additional tanks (85 l × 23 w × 34 h cm; net water volume 50 L). After a starvation period of 5 and 10 days, the sturgeons were video recorded in the same experimental tanks following the procedure described above (see Section 2.3). They were deprived of food for 5 days of starvation (DS) and then recorded. They were returned for another 5 DS (10 days in total) and recorded for the last time. After 10 DS, the tested fish were not returned to the husbandry tanks and were not repeatedly used. 

All video recordings were analysed with MATLAB software. To quantify the spatial distribution of the sturgeon within the experimental tank, each tank was subdivided by three horizontal lines into four equally sized rectangles and referred to as the 25% bottom, 50% middle, and 25% surface sections (see Figure 1). 

### 2.5. Statistical Analysis

To identify the effects of (a) two feeding applications (surface and bottom) with day and night feeding regimes and (b) 5 DS and 10 DS on the sturgeon’s swimming behaviour at the 25% bottom, 50% middle, or 25% surface section (See Figure 1) of the experimental tank, a pairwise comparison of the treatments was carried out using Fisher’s least square difference at a critical value (or confidence level) of *p* = 0.05. The possible bias that could emanate from replication was avoided by treating each experimental unit and having all the values averaged before they were used in the statistical analysis. The graphical models and statistical analysis were carried out using R statistical software (ggplot2′ package) [47]. The data are presented as mean ± SE. 

## 3. Results

### 3.1. Effect of Surface- and Bottom-Feeding Applications with Day and Night Regimes on Sturgeons Swimming Behaviour

#### 3.1.1. Effect of Daylight

**Figure 2:**
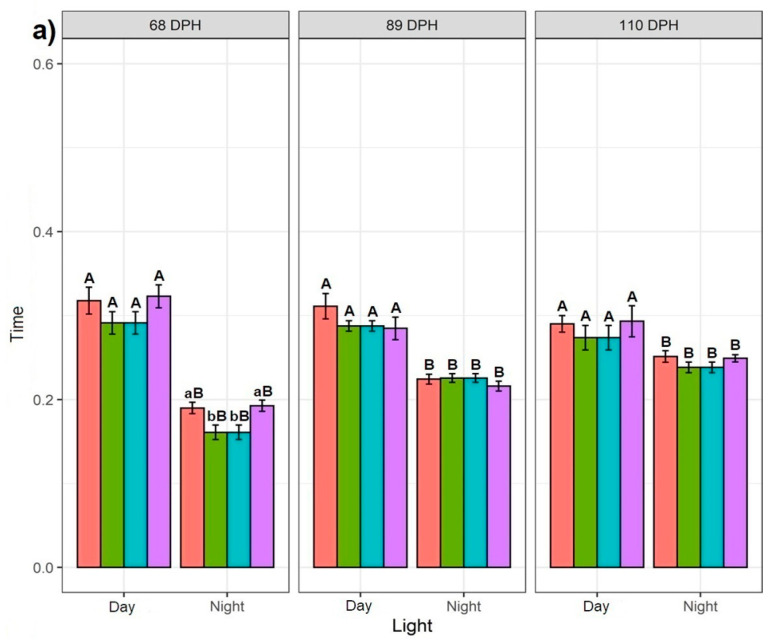

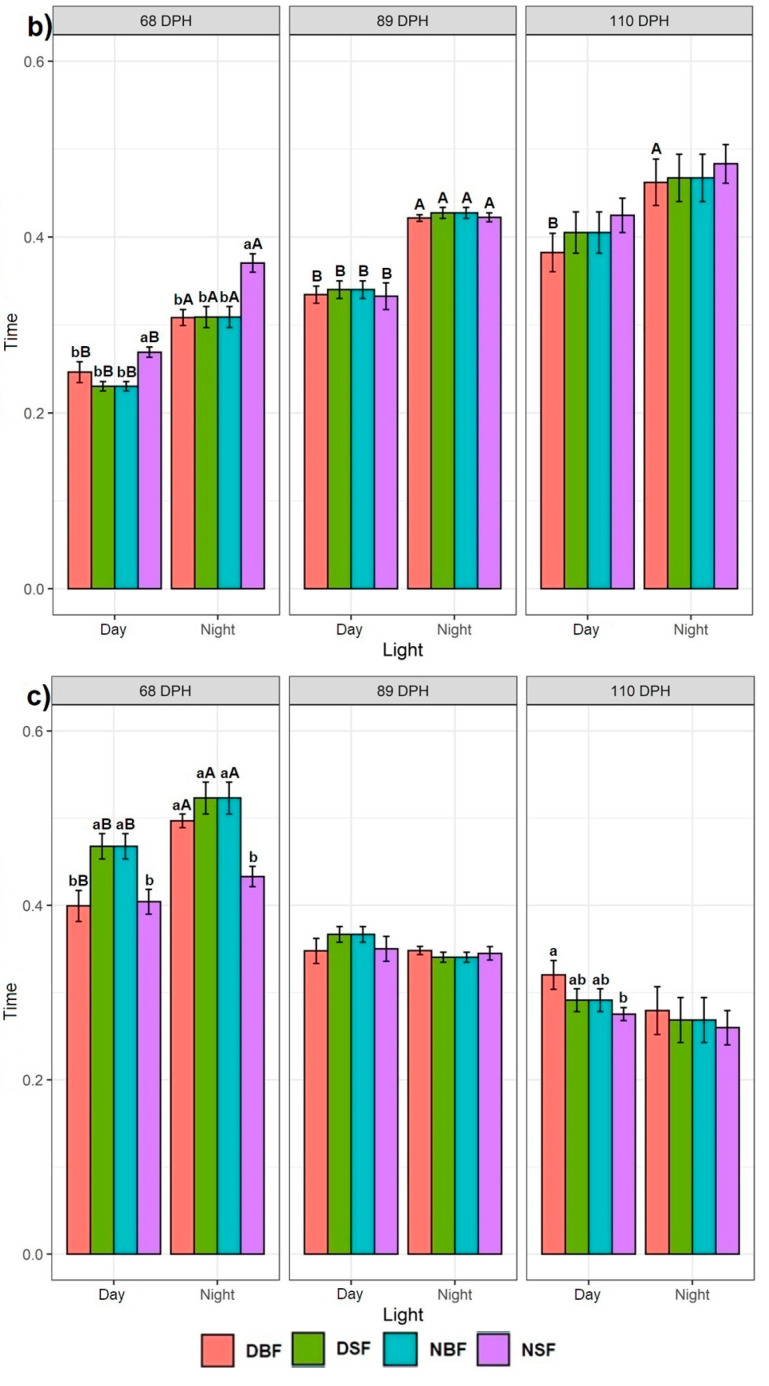
Effect of different feeding applications: DBF—day bottom feeding; DSF—day surface feeding; NBF—night bottom feeding; NSF—night surface feeding with day and night regimes on the Siberian sturgeon swimming preference: (**a**) at 25% bottom of the tank; (**b**) at 50% of the tank; (**c**) at 25% surface, which was recorded every three weeks at 68, 89 and 110 dph. Letters denote a significant difference, where the lowercase shows the difference between feeding applications for day or night regimes per recording day for each tank section. The uppercase letters show the difference for each feeding application between day and night per recording day for each tank section.

Regardless of the feeding application and time point, the fish spent significantly more time in the 25% bottom section of the tank during daylight than at night, while the difference between day and night decreased over consecutive time points (Figure 2a). This is reflected in the time spent in the 50% middle and 25% surface sections of the tank, where the 68 dph fish spent significantly more time during the night than the day (except for NSF at 68 dph in the 25% surface section of the tank where no difference was observed) (Figure 2c). Similarly, the 89 dph fish spent significantly more time in the 50% middle section of the tank during the night than day. However, no difference was observed in the time spent in the 25% surface section of the tank. The 110 dph fish showed no difference between the time spent during the day and night in both the 50% middle and 25% surface sections of the tank (except for DBF at 110 dph in the 50% middle section of the tank) (Figure 2b).

#### 3.1.2. Effect of Feeding Application

In general, the feeding application affected the time spent in particular parts of the tank only at 68 dph. The DBF and NSF fish at 68 dph spent more time in the 25% bottom section of the tank at night than the DSF and NBF fish (Figure 2a). A similar trend was observed in the 25% bottom section of the tank in daylight, although the result was not significant. The NSF fish at 68 dph spent significantly more time in the 50% middle section of the tank during the day and night than fish exposed to other feeding applications, which were not different. This was reflected in the 25% surface section of the tank, where NSF fish at 68 dph spent significantly less time during the night than other groups, whereas during the day, the NSF and DBF fish spent less time in this section than the NBF and DSF fish. No significant difference in feeding applications was observed among 89 and 110 dph fish (except for the significant difference in the time spent in the 25% surface section of the tank by 110 dph fish during the day) (Figure 2b,c).

### 3.2. Effect of Timepoints Recorded at 68, 89, and 110 dph on Sturgeons Swimming Behaviour

#### 3.2.1. Swimming Behaviour at One Particular Time Point

**Figure 3:**
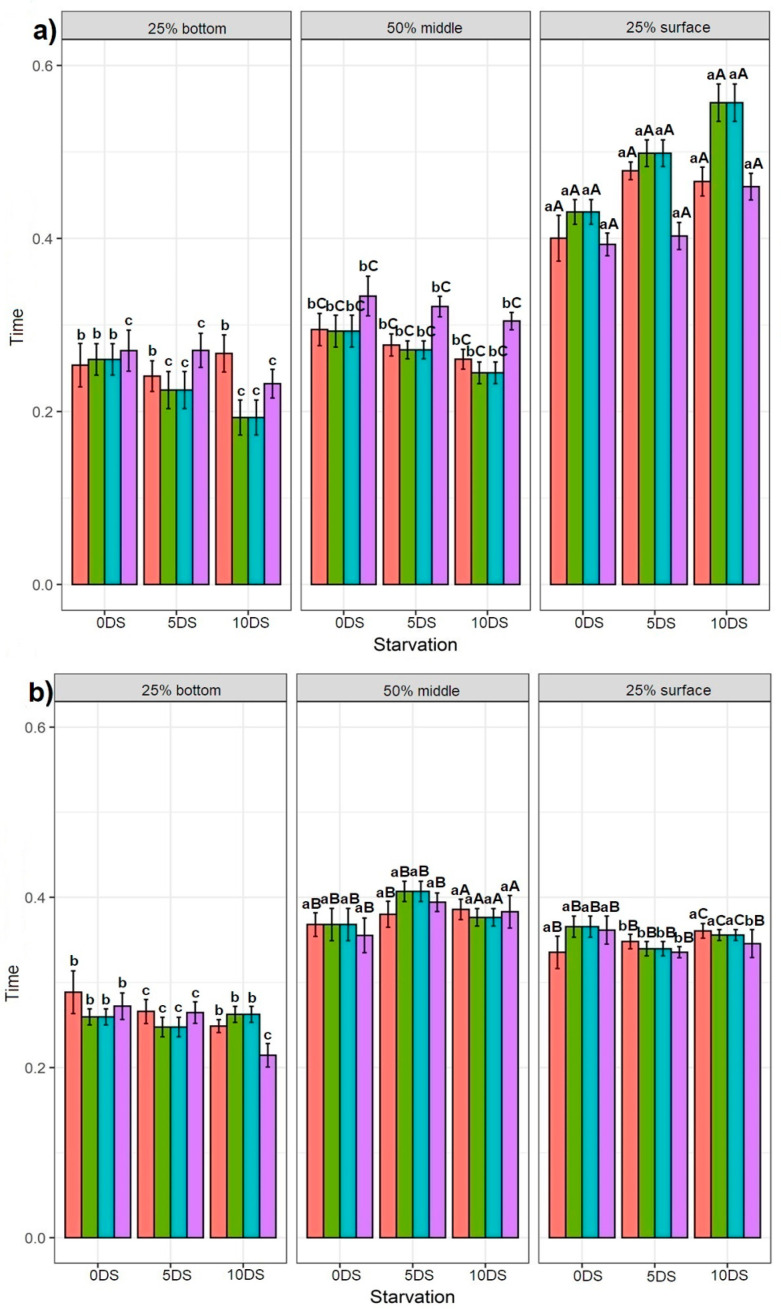

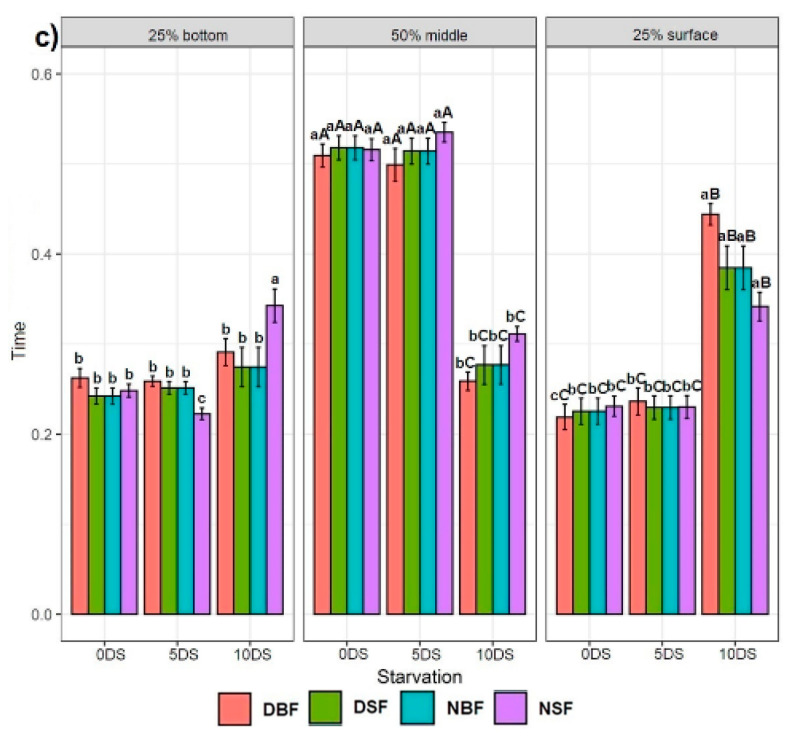
Effect of time points on the Siberian sturgeon swimming preference between three tank sections: (**a**) on the first recording day at 68 dph, (**b**) on the second recording day at 89 dph, and (**c**) on the third recording day at 110 dph. Letters denote significant differences, where the lowercase letters show the difference for each feeding application for one day of starvation between tank sections per recording day. The uppercase letters show the difference for each feeding application per one day of starvation for each tank section between three recording days.

In general, all fish groups spent significantly more time in the 25% surface section of the tank on recording day 68 (Figure 3a). Other tank sections showed that for 0 DS, the NSF fish spent significantly less time in the 25% bottom section than in the 50% middle section of the tank, while no difference was observed for BDF, DSF, and NBF fish. Regarding 5 and 10 DS, the DSF, NBF, and NSF fish spent significantly more time in the 50% middle section than in the 25% bottom section, with no difference observed in the DBF fish behaviour. On recording day 89, the highest swimming time spent in the 50% bottom and 25% surface sections of the tank was found in 0 DS fish (Figure 3b). A similar trend was observed in 10 DS fish (except for NSF, where no difference was found between the 25% bottom and 25% surface sections of the tank). Fish deprived for 5 DS spent significantly less time in the 25% bottom section of the tank, whereas the fish spent more time in the 50% middle section of the tank. The results of the 110th-day recording showed that fish deprived for 0 and 5 DS spent significantly more time in the 50% middle section of the tank than in the 25% bottom and 25% surface sections, in which the time spent was not different (Figure 3c). DBF, DSF, and NSF fish deprived for 10 DS spent significantly more time in the 25% surface section, although the time spent by the NSF group in the 25% surface and 25% bottom sections of the tank was not significantly different.

#### 3.2.2. Effect of Consecutive Time Points

In general, all fish groups spent significantly more time in the 50% middle and 25% surface sections of the tank, regardless of the time point of the recording (Figure 3). The fish deprived for 0, 5, and 10 DS showed no statistical difference in the time spent in the 25% bottom section of the tank during the 68th, 89th, and 110th recording days. For the 50% middle section of the tank, the fish deprived for 0 and 5 DS showed the highest time spent on day 110, while the fish deprived for 10 DS showed the highest time spent on day 89. Regardless of the days of starvation, the fish spent significantly more time in the 25% surface section of the tank on the 68th day. 

### 3.3. Effect of Feeding Application and 5 and 10 Days of Starvation on Sturgeons Swimming Behaviour

#### 3.3.1. Effect of Feeding Application on the Starved Fish

**Figure 4:**
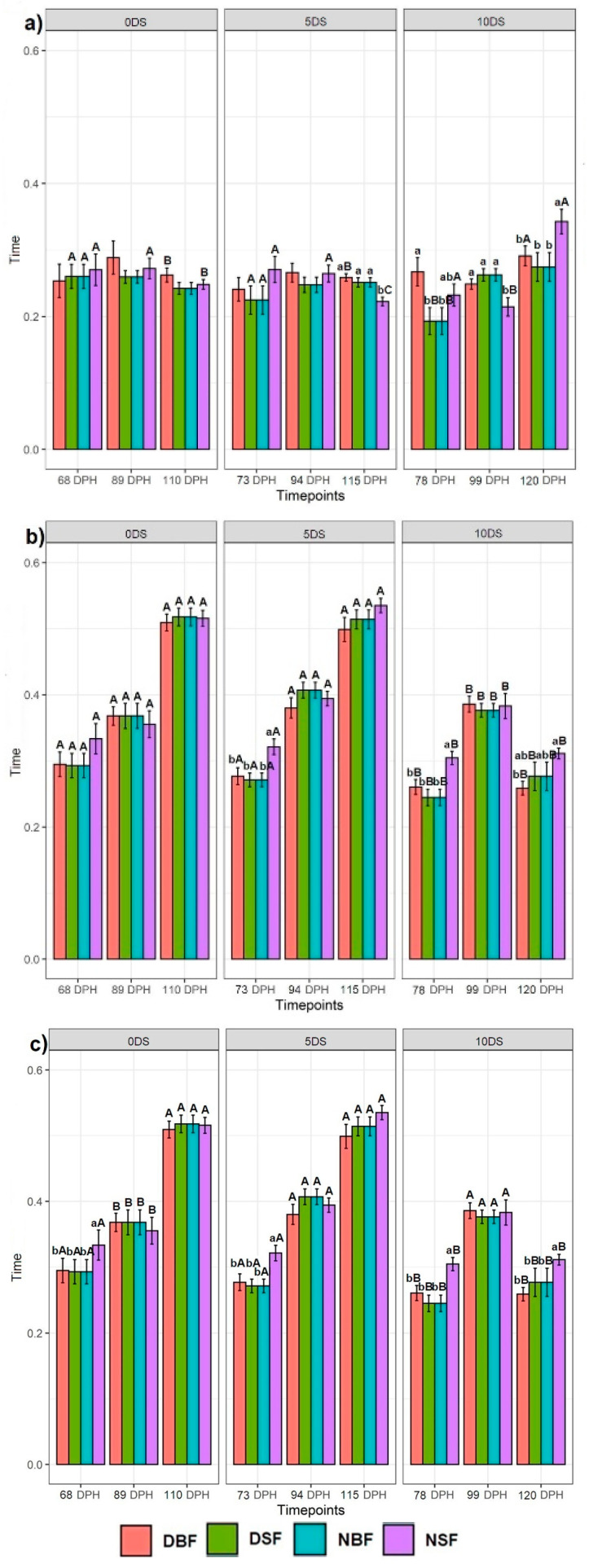
Effect of different feeding applications on the non-starved Siberian sturgeon (0 DS), starved for 5 days (5 DS), starved for 10 days (10 DS), and their swimming behaviour: (**a**) at the 25% bottom section of the tank; (**b**) at the 50% middle section of the tank; (**c**) at the 25% surface section of the tank. Letters denote significant differences, where the lowercase letters show the difference between feeding applications per recording day and per day of starvation for each tank section. The uppercase letters show the difference in each feeding application between 0, 5, and 10 days of starvation per tank section.

Feeding applications had no significant effect on the fish with 0 and 5 days of starvation in 25% bottom of the tank (except DBF, DSF, NBF fish on day 115 after being deprived for 5 DS, which spent significantly more time than NSF fish) (Figure 4a). The DSF and NBF fish deprived for 10 DS showed significantly less time spent in the 25% bottom section of the tank on day 78, while the NSF fish was not statistically different from fish exposed to the other feed applications. No significant difference was found between feed applications for fish deprived for 0 and 5 DS in the time spent in the 50% middle section of the tank (except on day 73 for fish deprived for 5 DS, where the NSF fish spent more time than the DBF, DSF, and NBF groups) (Figure 4b). Although we found no difference on day 99 in the time spent in the 50% middle section of the tank for all fish groups deprived for 10 DS, on days 78 and 120, the fish behaviour patterns were statistically different. On day 78, the NSF fish showed the highest time spent in the 50% middle section of the tank compared with the fish exposed to the other feeding applications (Figure 4b). On day 120, the DBF fish deprived for 10 DS showed significantly less time spent in the 50% middle section of the tank than NSF fish but was not different from DSF and NBF fish. The results obtained for the 25% surface section of the tank showed that there was no feeding application effect on all fish groups deprived for 0 and 5 DS, except on days 68 and 73, where the NSF fish spent more time in the 25% surface section of the tank than the fish exposed to the other feed applications (Figure 4c). On days 78 and 120, the NSF fish showed significantly more time spent in the 25% surface section of the tank than the rest, while no difference was found on day 99 among all feed application groups for fish deprived for 10 DS.

#### 3.3.2. Effect of 5 and 10 Days of Starvation on Sturgeon Behaviour

There was no clear trend in the effect of 5 and 10 DS on the sturgeon’s swimming behaviour in the 25% bottom section of the tank (Figure 4a). The DSF and NBF fish recorded on day 68 showed statistically less time spent in the 25% bottom section of the tank after 10 DS, while the DBF and NSF fish were not statistically different. The fish recorded on day 89 showed no statistical difference between 0, 5, and 10 DS (except the NSF fish, showing less time spent in the 25% bottom section of the tank after 10 DS). The DBF and NSF fish recorded on day 110 showed statistically more time spent in the 25% bottom section of the tank after 10 DS, while the DSF and NBF fish did not differ over time points (Figure 4a). We observed that 10 DS had a significant effect on the fish swimming behaviour in the 50% middle section of the tank, where the fish showed less time over three time points than the fish deprived for 0 and 5 DS, which were not statistically different (Figure 4b). A similar trend was observed for the 25% surface section of the tank (except for the fish groups recorded on 89 dph after 0 DS, which showed the lowest time spent in the 25% surface section of the tank) (Figure 4c).

## 4. Discussion

Restocking programmes of different fish species have been implemented worldwide [48,49,50,51]. However, the survival of hatchery-reared fish after release to riverine ecosystems is at a very low level [52]. One of the reasons for the high mortality rates of post-released fish is the modified swimming behaviour due to the hatchery rearing practice [51,53]. This was observed for Japanese flounders [39,41], sea trout parr [51], and may be expected for Siberian sturgeons as well. 

Generally, Siberian sturgeons are well adapted to benthic life. Juveniles do not typically swim up to the mid- and top-water layers. From hatching to 3 dph, wild Siberian sturgeon larvae swim up vertically and drift due to poorly developed fins. After 4 dph, their behaviour changes, and sturgeons become benthic with relatively low mobility [54,55]. The Siberian sturgeon reared in our recirculating aquaculture system at 89 and 110 dph showed a progressively higher preference for swimming in the middle part of the experimental tank. A previous study on Japanese flounders suggested that the surface feeding application promotes the frequent off-bottom swimming behaviour of benthic fish [56]. Although DSF, NBF, and DBF fish preferred the upper areas of the tank at the beginning of our experiment, our results did not highlight any striking difference between surface- and bottom-feeding applications. We also could not determine any impact of day/night light regimes on the swimming preference of the fish. We did not observe a similar spatial preference at later time points examined in the study, probably due to the periods of starvation and/or the predator-free environment.

Siberian sturgeons spent significantly more time in the upper tank after 5 and 10 DS in both day and night feeding groups. A higher level of swimming-up activity after 24 h of starvation was also reported for white sturgeon *Acipenser transmontanus* [57]. In our study, some sturgeons showed inverted swimming patterns (swimming with the ventral side upwards, see Figure S1), which may increase their susceptibility to predation in natural environments. This could be a hallmark of depleted and weakened fish. The inverted swimming pattern has already been documented in different sturgeon species, including in Siberian sturgeons, where the inverted swimming peaked between 8 and 32 dph [44,58]. This modified swimming behaviour was also observed in Japanese flounder, another benthic species, where fish jumped out of the water due to starvation just a few days after restocking [59]. This attentive behaviour was considered potentially lethal for fish since it increases the risk of being caught by predators [50,60]. The researchers in these previous studies have suggested that surface and inverted swimming behaviour represent adaptations to feeding at surfaces, but these assumptions probably do not explain why neither light regime nor feed application affected the periods of inverted swimming, as observed until the last day of our experiment. 

Another possible explanation for the progressive preference for swimming in the midwater column could be the predator-free environment of the rearing facility. The absence of predators most likely reduces the fish’s level of cautiousness and causes them to swim boldly to midwater column regions but deprives them of the foraging skills necessary for survival in the wild [61]. Previous studies documented an enhanced nocturnal swimming activity of wild early postlarval (10–20 dph) shovelnose sturgeon and shortnose sturgeon *Acipenser brevirostrum* in the upper water column, compared with the activity during daylight hours [44,62]. Higher foraging activity at night was also documented for postlarval lake sturgeon *Acipenser fulvescens* at the age of 42–69 dph [63], which are usually covered by nursery grounds during the day and are most likely to hide from predators. We also found the highest nocturnal activity in our sturgeons in the middle of the tank at the beginning of the experiments, regardless of the feeding applications. Later, the preference for swimming in the middle water column became less significant. A modified swimming behaviour due to anthropogenic husbandry was shown for various fish species [64,65,66], including brown trout [67] and Atlantic salmon [66]. The authors documented that one full hatchery-reared cycle was enough to induce maladaptive behaviour under natural conditions. This exposed the hatchery-reared generation to an increased risk of being caught by predators, which minimises the effectiveness of restocking programmes.

Several studies have reported that hatchery-reared fish can be taught to recognise potential predators before their return to natural habitats [40,68]. Training to recognise and respond to threat stimuli could be an efficient approach to improve the adaptation potential to natural conditions [30]. The exposure of hatchery-reared brook trout *Salvelinus fontinalis* to chemical stimuli from predatory chain pickerel *Esox niger*, coupled with alarm signals, demonstrated the learning of the fish to recognise the predator by decreased movement and altered foraging patterns [69]. The alarm and olfactory cues from predators were also investigated in the lake sturgeon and the Baltic sturgeon [63,70]. The sturgeons did not respond or only slightly did so to these stimuli, which were, however, reported to increase the general avoidance of predators [71]. The choice of predator used for the visual stimuli should be oriented towards the environment, where fish will be released. Dominant predators of the Caspian Sea are the northern pike *Esox lucius*, European perch *Perca fluviatiluis*, pikeperch *Sander lucioperca*, and catfish *Silurus glanis* [72,73,74,75] and may be considered for life skill training of sturgeons reared for the restocking of this environment. Learning applications involving multi-predators can significantly improve antipredator behaviour and increase the survival of hatchery-reared fish [76,77]. Furthermore, the application of small predators in trained predator recognition skills seems to be efficient [35]. To our knowledge, only a few studies have reported sturgeon’s life skills training with a one-predator approach [57,78], while multi-predator approaches remain largely unexplored. 

The age of fish seems to be a crucial parameter that influences the ability to learn predator recognition. For instance, Atlantic salmon aged 70–105 dph were more responsive to the presence of pike than their older conspecifics [79]. Age-depended learning skills to alarm cues were also reported for lake sturgeon [80,81]. These studies suggested that increased morphological development may lead to a decreased antipredator training response. Our findings suggest initiating life training skills before the age of 89 dph. 

## 5. Conclusions

The bottom-feeding application in the rearing facilities was expected to reduce the tendency of the Siberian sturgeon to swim up to the mid- and top-water layers after restocking. However, our results indicated that neither the type of feed application nor day/night feeding regimes had a significant impact on the swimming behaviour, rather the duration of starvation and the absence of predators. This observation may facilitate and improve the implementation of restocking programmes either through modified culture practices or the application of life skills training with predator stimuli.

## Data Availability

The data presented in this study are available in this manuscript and its supplementary materials and are available on request from the corresponding authors.

## Supplementary Materials

The following are available online at https://www.mdpi.com/article/10.3390/biology10111162/s1, Figure S1: Inverted swimming behaviour observed in our study.

## References

[1] Merino G., Barange M., Blanchard J.L., Harle J., Holmes R., Allen I., Allison E.H., Badjeck M.C., Dulvy N.K., Holt J. (2012). Can marine fisheries and aquaculture meet fish demand from a growing human population in a changing climate?. Glob. Environ. Chang..

[2] Duarte R., Pinilla V., Serrano A. (2014). Looking backward to look forward: Water use and economic growth from a long-term perspective. Appl. Econ..

[3] Food and Agriculture Organization of the United Nations (2017). The Future of Food and Agriculture—Trends and Challenges 2017.

[4] Shiklomanov I.A. (2000). Appraisal and assessment of world water resources. Water Int..

[5] Dudgeon D. (2000). Large-scale hydrological changes in tropical Asia: Prospects for riverine biodiversity: The construction of large dams will have an impact on the biodiversity of tropical Asian rivers and their associated wetlands. Bioscience.

[6] Nilsson C., Berggren K. (2000). Alterations of riparian ecosystems caused by river regulation: Dam operations have caused global-scale ecological changes in riparian ecosystems. How to protect river environments and human needs of rivers remains one of the most important questions of our time. Bioscience.

[7] Dudgeon D., Arthington A.H., Gessner M.O., Kawabata Z.-I., Knowler D.J., Lévêque C., Naiman R.J., Prieur-Richard A.-H., Soto D., Stiassny M.L. (2006). Freshwater biodiversity: Importance, threats, status and conservation challenges. Biol. Rev..

[8] Copp G.H. (1990). Effect of regulation on 0+ fish recruitment in the Great Ouse, a lowland river. Regul. Rivers Res. Manag..

[9] Jurajda P. (1995). Effect of channelization and regulation on fish recruitment in a flood plain river. Regul. Rivers Res. Manag..

[10] Haro A., Richkus W., Whalen K., Hoar A., Busch W.-D., Lary S., Brush T., Dixon D. (2000). Population decline of the American eel: Implications for research and management. Fisheries.

[11] Aarts B.G., Van Den Brink F.W., Nienhuis P.H. (2004). Habitat loss as the main cause of the slow recovery of fish faunas of regulated large rivers in Europe: The transversal floodplain gradient. River Res. Appl..

[12] Gao X., Brosse S., Chen Y., Lek S., Chang J. (2009). Effects of damming on population sustainability of Chinese sturgeon, *Acipenser sinensis*: Evaluation of optimal conservation measures. Environ. Biol. Fishes.

[13] Zhang H., Wei Q., Du H., Li L. (2011). Present status and risk for extinction of the Dabry’s sturgeon (*Acipenser dabryanus*) in the Yangtze River watershed: A concern for intensified rehabilitation needs. J. Appl. Ichthyol..

[14] Haxton T.J., Cano T.M. (2016). A global perspective of fragmentation on a declining taxon the sturgeon (Acipenseriformes). Endanger. Species Res..

[15] Lenhardt M.0, Jaric I., Kalauzi A., Cvijanovic G. (2006). Assessment of extinction risk and reasons for decline in sturgeon. Biodivers. Conserv..

[16] Bronzi P., Rosenthal H. (2014). Present and future sturgeon and caviar production and marketing: A global market overview. J. Appl. Ichthyol..

[17] Bronzi P., Rosenthal H., Gessner J. (2011). Global sturgeon aquaculture production: An overview. J. Appl. Ichthyol..

[18] Arndt G., Gessner J., Raymakers C. (2002). Trends in farming, trade and occurrence of native and exotic sturgeons in natural habitats in Central and Western Europe. J. Appl. Ichthyol..

[19] Knapp A., Kitschke C., Von Meibom S., Knapp A., Kitschke C., Von Meibom S. (2006). International Sturgeon Enforcement Workshop to Combat Illegal Trade in Caviar. Proceeding of International Sturgeon Enforcement Workshop to Combat Illegal Trade in Caviar, Brussels, Belgium, 27–29 June 2006.

[20] Navodaru I., Staraş M. (1998). Conservation of fish stocks in the Danube Delta, Romania: Present status, constraints, and recommendation. Ital. J. Zool..

[21] Williot P., Sabeau L., Gessner J., Arlati G., Bronzi P., Gulyas T., Berni P. (2001). Sturgeon farming in Western Europe: Recent developments and perspectives. Aquat. Living Resour..

[22] Wei Q., Zou Y., Li P., Li L. (2011). Sturgeon aquaculture in China: Progress, strategies and prospects assessed on the basis of nation-wide surveys (2007–2009). J. Appl. Ichthyol..

[23] Shen L., Shi Y., Zou Y., Zhou X., Wei Q. (2014). Sturgeon Aquaculture in China: Status, challenge and proposals based on nation-wide surveys of 2010–2012. J. Appl. Ichthyol..

[24] Brown C., Day R.L. (2002). The future of stock enhancements: Lessons for hatchery practice from conservation biology. Fish Fish..

[25] Ireland S.C., Beamesderfer R., Paragamian V., Wakkinen V., Siple J. (2002). Success of hatchery-reared juvenile white sturgeon (*Acipenser transmontanus*) following release in the Kootenai River, Idaho, USA. J. Appl. Ichthyol..

[26] Crossman J., Forsythe P., Scribner K., Baker E. (2011). Hatchery rearing environment and age affect survival and movements of stocked juvenile lake sturgeon. Fish. Manag. Ecol..

[27] Secor D., Houde E. (1998). Use of larval stocking in restoration of Chesapeake Bay striped bass. ICES J. Mar. Sci..

[28] Svåsand T., Kristiansen T.S., Pedersen T., Salvanes A.V., Engelsen R., Naevdal G., Nødtvedt M. (2000). The enhancement of cod stocks. Fish Fish..

[29] Myers R.A., Levin S.A., Lande R., James F.C., Murdoch W.W., Paine R.T. (2004). Hatcheries and endangered salmon. Science.

[30] Baer J., Blasel K., Diekmann M. (2007). Benefits of repeated stocking with adult, hatchery-reared brown trout, *Salmo trutta*, to recreational fisheries?. Fish. Manag. Ecol..

[31] Bell J.D., Bartley D.M., Lorenzen K., Loneragan N.R. (2006). Restocking and stock enhancement of coastal fisheries: Potential, problems and progress. Fish. Res..

[32] Saloniemi I., Jokikokko E., Kallio-Nyberg I., Jutila E., Pasanen P. (2004). Survival of reared and wild Atlantic salmon smolts: Size matters more in bad years. ICES J. Mar. Sci..

[33] Thorstad E.B., Uglem I., Arechavala-Lopez P., Økland F., Finstad B. (2011). Low survival of hatchery-released Atlantic salmon smolts during initial river and fjord migration. Boreal Environ. Res..

[34] Secor D., Arefjev V., Nikolaev A., Sharov A. (2000). Restoration of sturgeons: Lessons from the Caspian Sea sturgeon ranching programme. Fish Fish..

[35] Chebanov M., Rosenthal H., Gessner J., van Anrooy R., Doukakis P., Pourkazemi M., Williot P. (2011). Sturgeon Hatchery Practices and Management for Release Guidelines.

[36] McNeil W.J. (1991). Expansion of cultured Pacific salmon into marine ecosystems. Aquaculture.

[37] Salvanes A., Steele J.H., Thorpe S.A., Turekian K.K. (2010). 2001. Ocean ranching. Marine Policy and Economics.

[38] Blaxter J. (2000). The enhancement of marine fish stocks. Adv. Mar. Biol..

[39] Tanaka Y., Yamaguchi H., Gwak W.-S., Tominaga O., Tsusaki T., Tanaka M. (2005). Influence of mass release of hatchery-reared Japanese flounder on the feeding and growth of wild juveniles in a nursery ground in the Japan Sea. J. Exp. Mar. Biol. Ecol..

[40] Furuta S., Kitajima C. (1993). Releasing techniques and fry quality. Healthy Fry for Release, and Their Production Techniques.

[41] Miyazaki T., Masuda R., Furuta S., Tsukamoto K. (2000). Feeding behaviour of hatchery-reared juveniles of the Japanese flounder following a period of starvation. Aquaculture.

[42] Wishingrad V., Ferrari M.C., Chivers D.P. (2014). Behavioural and morphological defences in a fish with a complex antipredator phenotype. Anim. Behav..

[43] Nguyen R.M., Crocker C.E. (2006). The effects of substrate composition on foraging behavior and growth rate of larval green sturgeon, *Acipenser medirostris*. Environ. Biol. Fishes.

[44] Ross R., Bennett R. (1997). Comparative behaviour and dietary effects in early life phases of American sturgeons. Fish. Manag. Ecol..

[45] Chai Y., Tan F.X., Li L.X., Wei Q.W. (2014). Effects of delayed initial feeding on growth and survival of Chinese sturgeon (*Acipenser sinensis* Gray, 1835) larvae. J. Appl. Ichthyol..

[46] Hardy R.S., Zadmajid V., Butts I.A., Litvak M.K. (2021). Growth, survivorship, and predator avoidance capability of larval shortnose sturgeon (*Acipenser brevirostrum*) in response to delayed feeding. PLoS ONE.

[47] Wickham H., Chang W., Wickham M.H. (2016). Package ‘ggplot2’. Create Elegant Data Visualisations Using the Grammar of Graphics.

[48] Jokikokko E., Kallio-Nyberg I., Saloniemi I., Jutila E. (2006). The survival of semi-wild, wild and hatchery-reared Atlantic salmon smolts of the Simojoki River in the Baltic Sea. J. Fish. Biol..

[49] Czerniawski R., Pilecka-Rapacz M., Domagala J. (2011). Stocking experiment with Atlantic salmon and sea trout parr reared on either live prey or a pellet diet. J. Appl. Ichthyol..

[50] Tomiyama T., Watanabe M., Kawata G., Ebe K. (2011). Post-release feeding and growth of hatchery-reared Japanese flounder *Paralichthys olivaceus*: Relevance to stocking effectiveness. J. Fish. Biol..

[51] Krepski T., Czerniawski R. (2019). Can we teach a fish how to eat? The impact of bottom and surface feeding on survival and growth of hatchery-reared sea trout parr (*Salmo trutta trutta* L.) in the wild. PLoS ONE.

[52] Kellison G., Eggleston D., Burke J. (2000). Comparative behaviour and survival of hatchery-reared versus wild summer flounder (*Paralichthys dentatus*). Can. J. Fish. Aquat..

[53] Moberg O., Braithwaite V.A., Jensen K.H., Salvanes A.G.V. (2011). Effects of habitat enrichment and food availability on the foraging behaviour of juvenile Atlantic Cod (*Gadus morhua* L.). Environ. Biol. Fishes..

[54] Gisbert E. (1999). Early development and allometric growth patterns in Siberian sturgeon and their ecological significance. J. Fish. Biol..

[55] Gisbert E., Williot P., Castelló-Orvay F. (1999). Behavioural modifications in the early life stages of Siberian sturgeon (*Acipenser baerii*, Brandt). J. Appl. Ichthyol..

[56] Takahashi K., Masuda R., Yamashita Y. (2013). Bottom feeding and net chasing improve foraging behavior in hatchery-reared Japanese flounder *Paralichthys olivaceus* juveniles for stocking. Fish. Sci..

[57] Steel A., Hansen M., Cocherell D., Fangue N. (2019). Behavioral responses of juvenile white sturgeon (*Acipenser transmontanus*) to manipulations of nutritional state and predation risk. Environ. Biol. Fishes..

[58] Kasumyan A., Kazhlayev A. (1993). Formation of Searching Behavioral Reaction and Olfactory Sensitivity to Food Chemical Signals during Onlogeny of Sturgeons (Acipenseridae). J. Ichthyol..

[59] Tanaka M., Goto T., Tomiyama M., Sudo H. (1989). Immigration, settlement and mortality of flounder (*Paralichthys olivaceus*) larvae and juveniles in a nursery ground, Shijiki Bay, Japan. Neth. J. Sea Res..

[60] Furuta S. (1998). Comparison of feeding behavior of wild and hatchery-reared Japanese flounder, *Paralichthys olivaceus*, juveniles by laboratory experiments. Nippon Suisan Gakkaishi.

[61] Yamashita Y., Yamamoto K., Nagahora S., Igarashi K., Ishikawa Y., Sakuma O., Yamada H., Nakamoto Y. (1993). Predation by fishes on hatchery-raised Japanese flounder, *Paralichthys olivaceus*, fry in the coastal waters of Iwate prefecture, northeastern Japan. Aquac. Sci..

[62] Richmond A.M., Kynard B. (1995). Ontogenetic behavior of shortnose sturgeon, *Acipenser brevirostrum*. Copeia.

[63] Wishingrad V., Musgrove A.B., Chivers D.P., Ferrari M.C. (2015). Risk in a changing world: Environmental cues drive anti-predator behaviour in lake sturgeon (*Acipenser fulvescens*) in the absence of predators. Behaviour.

[64] Sundström L.F., Petersson E., Höjesjö J., Johnsson J.I., Järvi T. (2004). Hatchery selection promotes boldness in newly hatched brown trout (*Salmo trutta*): Implications for dominance. Behav. Ecol..

[65] Fernö A., Huse G., Jakobsen P.J., Kristiansen T.S., Nilsson J., Brown C., Laland K., Krause J. (2011). Fish behaviour, learning, aquaculture and fisheries. Fish Cognition and Behavior.

[66] Jackson C.D., Brown G.E. (2011). Differences in antipredator behaviour between wild and hatchery-reared juvenile Atlantic salmon (*Salmo salar*) under seminatural conditions. Can. J. Fish. Aquat..

[67] Alvarez D., Nicieza A. (2003). Predator avoidance behaviour in wild and hatchery-reared brown trout: The role of experience and domestication. J. Fish. Biol..

[68] Kawabata Y., Asami K., Kobayashi M., Sato T., Okuzawa K., Yamada H., Yoseda K., Arai N. (2011). Effect of shelter acclimation on the post-release movement and putative predation mortality of hatchery-reared black-spot tuskfish *Choerodon schoenleinii*, determined by acoustic telemetry. Fish. Sci..

[69] Mirza R.S., Chivers D.P. (2000). Predator-recognition training enhances survival of brook trout: Evidence from laboratory and field-enclosure studies. Can. J. Zool..

[70] Cámara Ruiz M., Espírito Santo C., Mai A., Gessner J., Wuertz S. (2019). Can juvenile Baltic sturgeon (*Acipenser oxyrinchus*) smell the enemy?. J. Appl. Ichthyol..

[71] Brown G.E., Ferrari M.C., Chivers D.P., Brown C., Laland K., Krause J. (2011). Learning about danger: Chemical alarm cues and threat-sensitive assessment of predation risk by fishes. Fish Cognition and Behavior.

[72] Lappalainen J., Dörner H., Wysujack K. (2003). Reproduction biology of pikeperch (*Sander lucioperca* (L.))—A review. Ecol. Freshw. Fish.

[73] Komsari M.S., Bani A., Khara H. (2015). Growth and population structure of the European perch, *Perca fluviatilis* Linnaeus, 1758 (Osteichthyes: Percidae) in the Anzali Wetland south-west Caspian Sea. Indian J. Fish..

[74] Khara H., Sattari M. (2016). Occurrence and intensity of parasites in Wels catfish, *Silurus glanis* L. 1758 from Amirkelayeh wetland, southwest of the Caspian Sea. J. Parasit. Dis..

[75] Ebrahimzadeh Kouchesfahani N., Forouhar Vajargah M. (2021). A short review on the biological characteristics of the species *Esox lucius*, Linnaeus, 1758 in Caspian Sea Basin (Iran). Transylv. Rev. Syst. Ecol. Res..

[76] Templeton C.N., Shriner W.M. (2004). Multiple selection pressures influence Trinidadian guppy (*Poecilia reticulata*) antipredator behavior. Behav. Ecol..

[77] Darwish T.L., Mirza R.S., Leduc A.O., Brown G.E. (2005). Acquired recognition of novel predator odour cocktails by juvenile glowlight tetras. Anim. Behav..

[78] Hintz W., Grimes G., Garvey J. (2013). Shovelnose sturgeon exhibit predator avoidance behaviour in the presence of a hungry predator. J. Appl. Ichthyol..

[79] Hawkins L.A., Magurran A.E., Armstrong J.D. (2008). Ontogenetic learning of predator recognition in hatchery-reared Atlantic salmon, *Salmo salar*. Anim. Behav..

[80] Sloychuk J.R., Chivers D.P., Ferrari M.C. (2016). Juvenile lake sturgeon go to school: Life-skills training for hatchery fish. Trans. Am. Fish. Soc..

[81] Wishingrad V., Sloychuk J., Ferrari M., Chivers D. (2014). Alarm cues in Lake Sturgeon *Acipenser fulvescens* Rafinesque, 1817: Potential implications for life-skills training. J. Appl. Ichthyol..

