# Supplementary figures and images for: The Effect of Different Feeding Applications on the Swimming Behaviour of Siberian Sturgeon: A Method for Improving Restocking Programmes
Source: Biology (Basel). 2021 Nov 10;10(11):1162. doi: 10.3390/biology10111162 (PMC8614938; doi:10.3390/biology10111162)

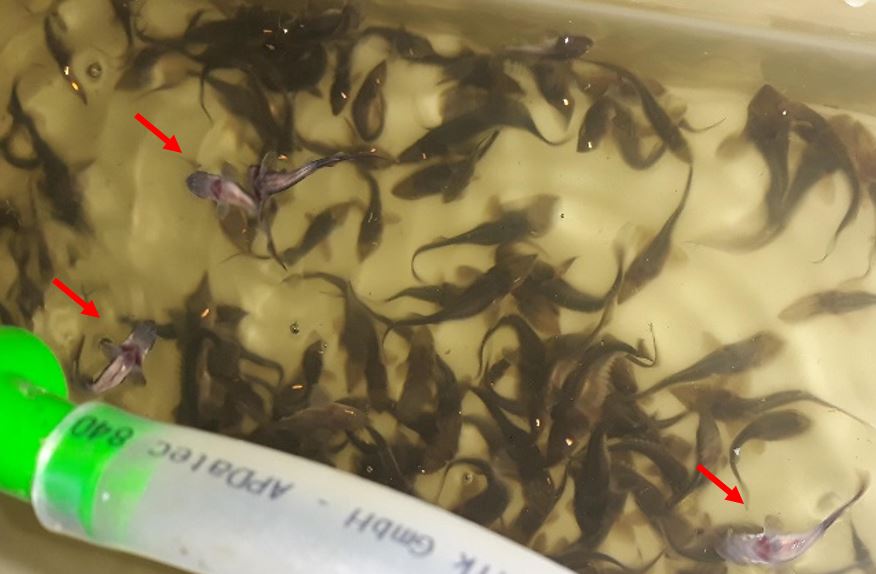

Supplement: Supplementary file 1 [file biology-10-01162-s001.zip › biology-1442444-supplementary.JPG]
